# Leveraging AI for Analysis of Digital Health Information on Cancer Prevention Among Arab Youth and Adults: Content Analysis

**DOI:** 10.2196/77888

**Published:** 2026-02-09

**Authors:** Alia Komsany, Obada Al Zoubi, Laetitia Sebaaly, Gabrielle Harrison, Orysya Soroka, Safa ElKefi, David Scales, Erica Phillips, Laura C Pinheiro, Israa Ismail, Perla Chebli

**Affiliations:** 1 Division of General Internal Medicine Weill Cornell Medicine New York City, NY United States; 2 Independent Researcher Boston, MA United States; 3 Independent Consultant New York, NY United States; 4 School of System Sciences and Industrial Engineering Watson College of Engineering Binghamton University New York, NY United States; 5 NYU Langone Health New York City, NY United States

**Keywords:** AI-driven content analysis, cancer prevention, engagement, digital health communication, TikTok

## Abstract

**Background:**

As TikTok (ByteDance) grows as a major platform for health information, the quality and accuracy of Arabic-language cancer prevention content remain unknown. Limited access to culturally relevant and evidence-based information may exacerbate disparities in cancer knowledge and prevention behaviors. Although large language models offer scalable approaches for analyzing online health content, their utility for short-form video data, especially in underrepresented languages, has not been well established.

**Objective:**

We aimed to characterize and evaluate the quality of Arabic-language TikTok videos on cancer prevention and explore the use of large language models for scalable content analysis.

**Methods:**

We used the TikTok research application programming interface and a GPT-assisted keyword strategy to collect Arabic-language TikTok videos (2021-2024). From an initial collection of 1800 TikTok videos, 320 were eligible after preprocessing. Of these, the top 25% (N=30) most-viewed were analyzed and manually coded for content type, cancer type, uploader identity, tone and register, scientific citation, and disclaimers. Video quality was assessed using the Patient Education Materials Assessment Tool for Audiovisual Materials for understandability and actionability, and the Global Quality Scale (GQS). GPT-4 was used to generate artificial intelligence annotations, which were compared to human coding for select variables.

**Results:**

The top 25% (N=30) most-viewed videos amassed a total of 21.6 million views. Diet and alternative therapies were most common (15/30, 50%), which included recommendations to reduce hydrogenated oils, increase fruit and vegetable intake, and the use of traditional remedies such as garlic and black seed. Only 6.6% (2/30) of videos cited scientific literature. General cancer (15/30, 53%), breast (5/30, 17%), and cervical (4/30, 13%) cancers were most frequently mentioned. Doctors led 30% (9/30) of videos and were more likely to produce higher quality content, including significantly higher global quality scores (GQS=4, median 4, IQR 4-4 vs 3, median 3, IQR 2-3, P=.06). Over half of the videos had low understandability (16/30, 53%) and actionability (18/30, 60%). Emotionally framed content had the highest engagement across likes and shares, although this did not reach statistical significance (P=.08 and P=.05, respectively). However, emotional tone was significantly associated with lower GQS scores (P=.01). GPT-4 showed high agreement with human coders for cancer type (Cohen κ=1.0), strong agreement for GQS (κ=0.94), but low agreement for tone classification (κ=0.15), due to misclassification of emotional delivery from text-only input.

**Conclusions:**

Arabic-language TikTok cancer prevention content is highly engaging but variable in quality, with emotionally framed videos attracting substantial attention despite lower informational value. Artificial intelligence-assisted tools show strong potential for scalable, multilingual health content analysis, but multimodal approaches are needed to accurately interpret tonal and audiovisual features.

## Introduction

### Global Burden of Cancer

Worldwide, the cancer burden continues to rise, with an estimated 20 million new cases and 9.7 million deaths reported in 2022, projected to reach 35 million cases by 2050 [1]. The Arab world, which includes 22 countries, faces a particularly rapid increase in cancer incidence, with rates expected to rise 1.8-fold by 2030 [2]. Cancer ranks as a leading cause of death in many Arab nations, with Lebanon reporting the highest incidence of bladder cancer worldwide and Egypt contributing significantly to global liver cancer mortality [3,4]. Despite these rising trends, cancer prevention awareness remains limited, with low participation in screening programs and persistent misconceptions about cancer causes and treatment [5-8].

### TikTok as a Source of Health Information

Social media platforms, such as TikTok, an emerging short-video app, have become major sources of health information worldwide [9]. During the COVID-19 pandemic, the platform saw a surge in health professionals and organizations using it to share medical knowledge and public health messages [10]. This shift highlighted the growing need for health care professionals to integrate video-based social media platforms, such as TikTok, into digital health communication strategies [11]. However, TikTok’s global reach comes with region-specific challenges. Unlike other US-based platforms that apply universal moderation policies, TikTok uses localized moderation, tailoring its policies by region. This has raised concerns among Arabic-speaking users, particularly in North Africa, where dialect-specific moderation tools are lacking. Users often resort to strategies such as “algospeak” to avoid perceived censorship, and content moderation algorithms developed with limited dialect training data and nonnative annotators may misclassify or fail to flag harmful health misinformation. These dynamics highlight the urgent need to ensure equitable, culturally sensitive content governance as platforms such as TikTok become central to health communication ecosystems [12].

### Arabic-Speaking Populations, an Understudied Demographic

Arabic-speaking populations, both in Arab countries and in diaspora communities, represent an understudied demographic in cancer prevention research [13,14]. Cultural beliefs, religious considerations, and misinformation often influence health behaviors, contributing to lower participation in preventive measures [15,16]. Language barriers further restrict access to reliable health information, with the Arabic language notably underrepresented in digital health research, along with the scarcity of validated Arabic-language health literacy tools and medical datasets [17-19]. This lack of research makes it challenging to assess the accuracy and effectiveness of Arabic-language health content, particularly on social media [20-22]. Understanding how cancer prevention messages are framed, their alignment with evidence-based guidelines, and their audience engagement is crucial for improving digital health communication among Arab nations and diaspora populations [23,24].

### Large Language Models for Analyzing Digital Health Communication

Advancements in artificial intelligence (AI), particularly large language models (LLMs) such as GPT, offer new opportunities for analyzing digital health communication in understudied languages [25,26]. GPT-4 has demonstrated high accuracy in detecting sentiment, misinformation, and medical accuracy across multiple languages [26]. Unlike traditional natural language processing tools, GPT does not require extensive language-specific training, making it a scalable tool for content analysis [26-28]. This study sought to examine TikTok videos on cancer prevention in Arabic, assess the content quality of the videos, and explore the role of LLMs such as GPT-4 in evaluating digital health content. By identifying gaps in digital health communication, this research seeks to inform strategies for improving cancer prevention awareness among Arabic-speaking communities.

## Methods

### Data Source and Retrieval

**Figure 1:**
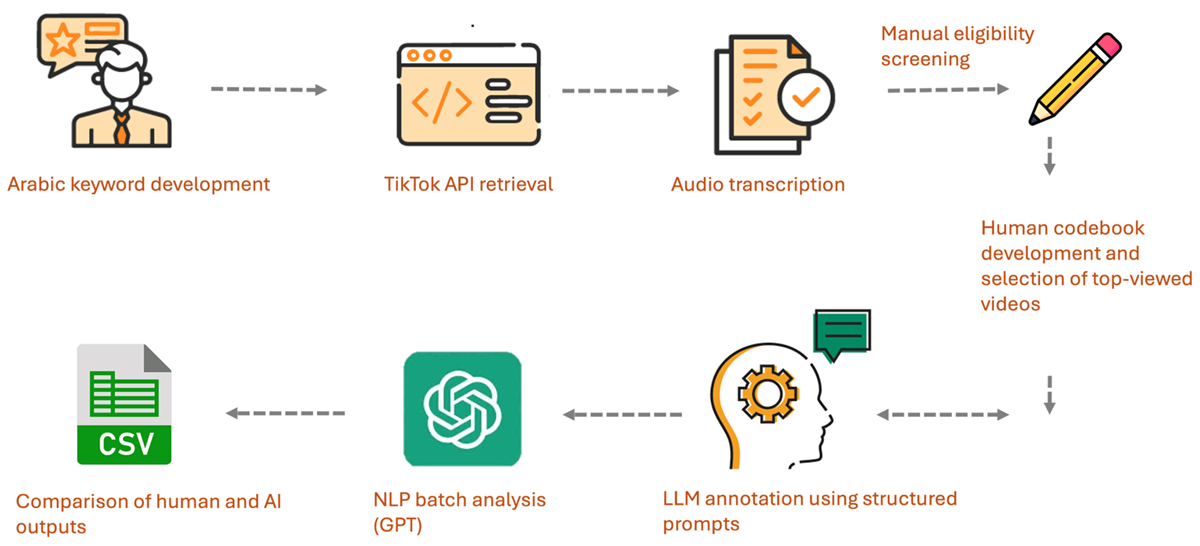
Overview of the analytic workflow: Arabic-language TikTok videos were retrieved using an iterative keyword strategy, transcribed, and screened for eligibility. A subset was used to develop the coding framework, after which the top 25% (N=30) most-viewed videos were manually coded and annotated using a large language model. Agreement between human and AI classifications was assessed using Cohen κ coefficient. Full methodological details are provided in the Methods section. AI: artificial intelligence; API: application programming interface; LLM: large language model; NLP: natural language processing.

We used a multistep analytic workflow to identify, process, and analyze Arabic-language TikTok videos related to cancer prevention, integrating human coding with AI-assisted annotation. Figure 1 provides an overview of the full workflow, including keyword development, video retrieval, transcription, eligibility screening, manual coding, AI-based annotation, and assessment of agreement between human and AI outputs.

Using the TikTok application programming interface (API), we retrieved Arabic-language TikTok videos related to cancer prevention and the HPV vaccine from 2021 to 2024. This time frame was selected based on data from the Arab Youth Survey, which indicated an increasing trend in TikTok market penetration among young Arabs aged 18 to 24 years during this period. Specifically, daily TikTok usage more than doubled from 21% in 2020 to 50% in 2022, highlighting the platform’s growing influence during this period [29]. Given that younger generations often play a key role in disseminating health information within their families, this period was considered optimal for capturing relevant content.

### Search Strategy and Transcription

**Figure 2:**
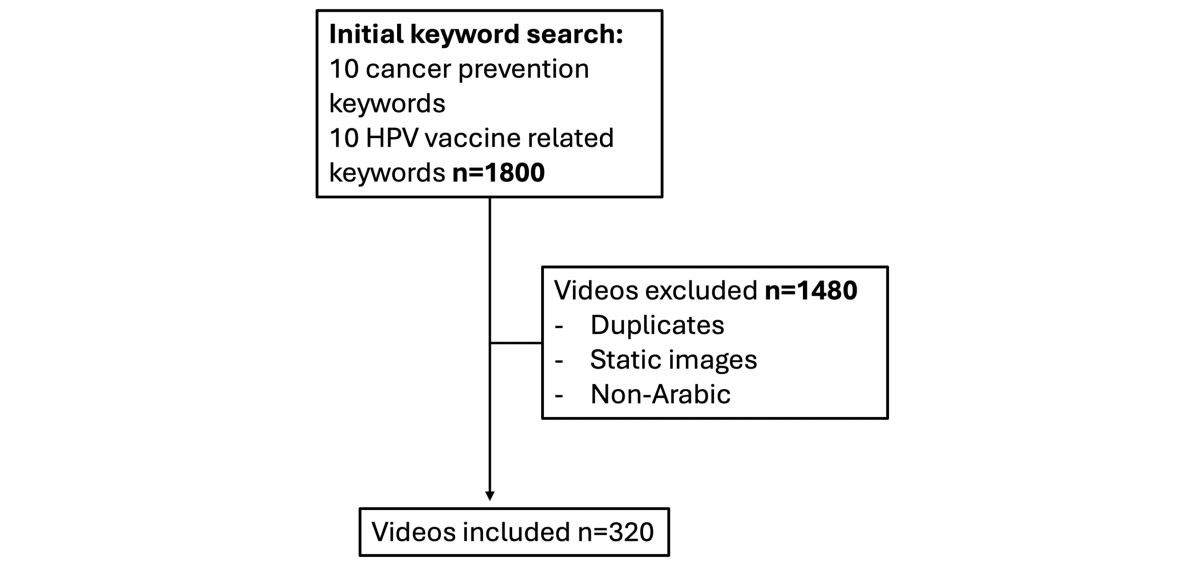
The filtering process used to identify top-performing Arabic-language TikTok videos related to cancer prevention. The final dataset included 30 videos in the top 25% most-viewed videos.

The research team developed an Arabic keyword (20 keywords) list focused on cancer prevention and HPV vaccination (Figure 1), which was iteratively refined and expanded using GPT-4 to ensure broad topical coverage (eg, “cancer prevention,” “HPV vaccine,” “tumor prevention,” “healthy nutrition to prevent cancer,” and “vaccination to protect against cervical cancer”). We then collected 1800 videos as above, excluding captions and comments (Figure 2). Videos were retrieved over several days because we were limited to 10,000 requests every 24 hours. Videos were transcribed using Sonix AI, selecting the Arabic transcription option. This Arabic transcription service is designed to handle a wide range of accents and regional dialects using advanced speech-to-text technology, though the model cannot be fine-tuned by users. All transcripts were manually reviewed for accuracy by the first author. No videos were truncated or cut during transcription, and the full audio of each TikTok was captured by Sonix’s Arabic transcription. Sonix transcriptions focused on the lexical content of speech, the words and their semantic meaning, rather than acoustic properties such as pitch, pauses, or emphasis. As a result, nonverbal or visual information was not represented.

As part of our initial data collection using the TikTok API, we also retrieved publicly available aggregate engagement metrics for each video by country. These data, though not directly linked to the final analytic sample, provided insight into the geographic reach and broader engagement with Arabic-language cancer prevention content across global audiences. This included data on total views, likes, and shares by country, allowing us to assess which regions exhibited the highest levels of user interaction.

### Codebook Development Using a Random 50-Video Subset

A random 16% (n=50) subset of the 320 videos was manually coded to create and refine the codebook. The research team reviewed coded data, discussed emerging categories, and reached consensus on definitions. The final codebook included classifications for content type, cancer type mentioned, tone and register, uploader type, presence of religious references, cautionary messages or disclaimers, target demographic, content quality indicators (global quality score and the Patient Education Materials Assessment Tool for Audiovisual Material [PEMAT AV]). Videos were coded as referencing scientific literature if they included direct citations or explicit mentions of scientific guidelines. This was used as a proxy for transparency, not as a definitive assessment of evidence-based accuracy. Tone and register were coded and simplified to 2 dimensions: emotional tone (eg, personal storytelling or emotional appeal, inclusive of expressive vocal delivery such as raised voice and dramatic pauses) and linguistic register (eg, casual or serious). This enabled consistency in both manual coding and AI-based classification and prioritized standardization over nuanced qualitative distinctions. If multiple tones were present within a video, the dominant style was recorded based on overall delivery. Videos were also coded for the specific types of cancer mentioned, including breast, colorectal, liver, pancreatic, brain, lung, cervical, oral, bladder, lymphoma, prostate, other cancers, and general cancer content (Textbox 1).

**Descriptions of content features and coding categories applied to Arabic TikTok videos.**

**Evidence-based**

- Reference to a scientific study or cites scientific literature.

**Emotional tone and linguistic register**

- Describes how information is delivered:
  Emotional tone: expresses personal experiences, urgency, or affective delivery. For example, “Dealing with this person causes cancer… it’s not food or drink that harms you; it’s people.”
  Linguistic register: Casual: uses everyday, friendly language. For example, “I wish we could change this behavior, because it's literally a fountain of cancerous tumors.” Serious: uses formal or urgent phrasing. For example, “You have to come to the clinic. We have to do the mammogram. We have to take a sample. We have to..”

**Content types**

- Cancer prevention topics mentioned:
  Diet alternative therapies: content promoting natural or nonclinical cancer prevention strategies, including the use of raw garlic, black seed, and dietary recommendations such as reducing hydrogenated oils.
  Screening and early detection: mammograms and Papanicolaou test smears.
  Vaccination: HPV vaccine.
  Self-examination and symptom awareness: breast or testicular checks.
  Smoking cessation: avoiding tobacco.
  Stress or negativity: links between stress and cancer.
  Survivor experience: sharing stories of cancer survival.
  Chemical carcinogens: mention of environmental or food-based chemicals.

**Cancers mentioned**

- Specific and nonspecific cancer types cited, including:
  General, breast, colorectal, liver, pancreatic, brain, lung, cervical, oral, bladder, lymphoma, prostate, other, and no cancer mentioned.

**Speaker (doctor, self ID, or layperson)**

- Whether the speaker self-identifies as a doctor (based on credentials in profile or linked accounts), self-identifies without affiliation, or does not claim any medical background.

**Religious reference**

- Mentions religious texts or spiritual framing of health advice.

**Cautionary message or disclaimer**

- Debunks a myth, adds a disclaimer, or highlights risks.

**Target demographic**

- Intended audience includes women, men, both genders, and youth.

**Patient Education Materials Assessment Tool (PEMAT) understandability**

- Percentage score based on clarity and ease of understanding.
- Coded as: high: 67%-100%, medium: 34%-66%, and low: 0%-33%.

**PEMAT actionability**

- Percentage score based on clear steps for action. Same categorization as understandability.

**Global Quality Scale (GQS)**

- 1=very poor (not useful) to 5=excellent (highly useful and comprehensive).

The coding framework included an assessment of uploader type which was classified into three categories: (1) doctors, whose credentials were corroborated through profile information (eg, “Dr” in username or bio); (2) self-identified doctors, who claimed a medical background without confirmable credentials; and (3) laypersons, with no stated or apparent medical affiliation. When the uploader status was ambiguous, the research team reviewed TikTok biographies, posted video content, and any linked social media profiles to determine affiliation. Clinic-affiliated accounts with a medical focus were also coded as doctor-led. Influencer status was assessed separately based on follower count, due to limited public information or unidentifiable handles. Creators with 100,000 or more followers were classified as influencers, regardless of medical background or professional identity. Videos were also coded for references to religious texts or beliefs, including phrases that framed health outcomes as divinely guided (*qadr*). Cautionary messages were defined as explicit statements that debunk myths, provide disclaimers, or warn against specific risks, such as ensuring that individuals with specific conditions avoid potential harms associated with alternative therapies or clarifying that the HPV vaccine is not exclusively for girls.

The target demographic of each TikTok video was categorized based on direct mention of the audience in the video, such as if the creator explicitly addressed a specific group (women or men), the reference to a specific age group (young people), or broad health advice, which was categorized as both genders.

### Sample Selection Based on the 75th Percentile Cutoff

To focus on the most visible content, we selected the top 25% (N=30) most-viewed videos for detailed manual and AI analysis. To identify the most engaged content, we applied a 75th percentile cutoff. The decision to use the 75th percentile was based on established statistical principles for performance classification, a method that aligns with industry standards and prior studies that have used percentile-based cutoffs (75th percentile) to differentiate high-performing content from lower-engagement material [30]. This method helps mitigate the influence of outliers while allowing for the analysis of content that drives interaction and user engagement. By applying this approach, we ensured that this subset of videos reflected the most influential cancer prevention messaging on TikTok, aligning with established research methodologies in social media health communication [30].

### Manual Coding and Interreliability Testing

The coding of the top 25% (N=30) of most-viewed videos was independently conducted by 2 study team members (AK and LS). The 2 coders met to finalize the codebook and resolve any coding discrepancies. To ensure coding reliability, interrater agreement was assessed using Cohen κ. Cohen **κ** was calculated for each coding category before reconciliation. Cohen **κ** values were interpreted using the following standard: values below 0.20 indicated slight agreement; 0.21-0.40, fair agreement; 0.41-0.60, moderate agreement; 0.61-0.80, substantial agreement; and 0.81-1.00, almost perfect agreement.

### Assessment of Understandability, Actionability, and Quality

The PEMAT AV was used to assess the understandability and actionability of the videos [31]. The understandability section contains 13 items, and the actionability section includes 4 items, which can each be scored as 0 (“disagree”), 1 (“agree”), or “not applicable.” For each section, PEMAT AV scores are calculated as percentages by dividing the points achieved by the items evaluated for the video. Therefore, higher values are indicative of higher understandability and actionability. The PEMAT AV has been widely used to evaluate health communication materials across formats, including videos, animations, and patient education modules for a range of topics, including chronic disease management, vaccine education, cancer prevention, and health literacy interventions [32,33]. We dichotomized PEMAT AV scores with 0%-66% considered as “low understandability,” and 67%-100% as “high understandability.” This threshold was based on the original guidance provided by Shoemaker et al [31], who recommended 70% as a benchmark for acceptable educational materials. The Global Quality Scale (GQS) was used to evaluate the overall quality, flow, and usefulness of each video’s health information. This 5-point Likert scale has been widely used to evaluate the reliability and educational value of online medical and public health content [34]. The score represents the perception of the trained coder (in our case, 2 Arabic-speaking coders with experience evaluating health communication content). The GQS is scored based on the following scale 1=“very poor quality, missing information, not useful”; 2=“generally poor quality, some missing information, very limited use”; 3=“moderate quality, some information adequately discussed, somewhat useful”; 4=“good quality, most relevant information discussed, useful”; and 5=“excellent quality, all relevant information discussed, very useful” [35].

### AI-Based Annotation

To generate AI-based annotations, we used a 1-shot prompting approach, in which a single, structured prompt was provided to the model to classify each video based on predefined categories from our manually generated codebook [36]. The prompt included clear definitions to guide the model’s interpretation. For instance, the model was instructed as follows: “Answer the questions as precisely and faithfully as possible using the provided context. The provided text is in Arabic with various dialects. Provide the answers in JSON format. Ensure that all responses are directly based on the provided text without assumptions or external information.”

Questions included items such as “List any cancers mentioned in the text: Options: General, Breast, Colorectal, Liver, Pancreatic, Brain, Lung, Cervical, Oral, Bladder, Lymphoma, Prostate, Other and No cancer mentioned.” For GQS scoring: “A Global Quality Score (GQS): Options: a score from 1 to 5 with:1: Poor quality, poor flow, and not useful 2: Generally poor quality and flow, but some information is listed 3: Moderate quality and flow, but some important information is poorly discussed, 4: Good quality and flow, but some topics are not covered 5: Excellent quality and flow, and very useful.”

For each coding category, such as cancer type or GQS score, the model was instructed to return 1 label per video, such as output the answers in the following JSON format: “cancers_mentioned”: [<list from cancers list>], “GQS_Score”: “<one score>”

AI outputs were generated in Python (Python Software Foundation) through a batch analysis pipeline [37]. The GPT model was optimized using iterative prompt engineering, refining the input structure to improve consistency in classification and fidelity to the codebook. This enabled efficient, scalable annotation of video characteristics while minimizing ambiguity, and facilitated direct comparison between AI-generated and human-coded classifications. AI-generated outputs were reviewed by human coders and systematically compared to manual annotations for the top-viewed videos. Cohen **κ** was used to evaluate interrater reliability between human and AI classifications. This analysis was conducted using the Cohen kappa score function in Python, which measures agreement beyond chance for categorical variables.

### Statistical Analysis

As this was an exploratory analysis, GPT-generated annotations were limited to 3 key categories: cancer type, tone and register, and GQS score. Descriptive statistics were used to summarize video characteristics, and inferential analyses were conducted using nonparametric tests due to high variability in the data. The Wilcoxon Rank Sum test was used to compare median engagement metrics (likes and shares) across groups, as engagement data were highly skewed. Fisher Exact Tests were used for categorical comparisons where sample sizes were small or expected cell counts were low. These statistical methods were selected to ensure robustness despite nonnormal distributions and heterogeneous group sizes.

### Ethical Considerations

This study analyzed publicly available TikTok videos related to cancer prevention using the TikTok Research API and did not involve direct interaction with human participants. No private, identifiable, or nonpublic user data were collected. All data were accessed and analyzed in accordance with TikTok’s terms of service and research data use policies.

## Results

### Overview

The final analytic dataset included 30 TikTok videos, representing the top 25% most-viewed content from an initial pool of 320 Arabic-language videos related to cancer prevention (cutoff at 59,640 views). These 30 videos collectively amassed 21.6 million views, 445,000 likes, and 146,000 shares (see Tables 1 and 2).

**Table 1:** Characteristics of TikTok videos (N=30).

Characteristic		Values, n (%)
**Content type**		
	Diet and alternative therapies	15 (50)
	Screening and early detection	6 (20)
	HPV^a^ vaccination	3 (10)
	Self-examination and symptoms to look out for	1 (3)
	Smoking cessation	1 (3)
	Stress and negativity	1 (3)
	Survivor experience	1 (3)
	Chemical carcinogens	1 (3)
**Cancers mentioned**		
	General cancer	15 (50)
	Breast cancer	6 (20)
	Cervical cancer	4 (13)
	Colon cancer	2 (7)
	Bladder cancer	1 (3)
	Multiple cancers	1 (3)
	Testicular cancer	1 (3)
**Emotional tone and register**		
	Casual	16 (53)
	Serious	8 (27)
	Emotional	6 (20)
**Target demographic**		
	Both genders	19 (63)
	Women	8 (27)
	Men	1 (3)
	Young people	2 (7)
**Led by doctors (corroborated or self-identified)**		
	Yes (credentials corroborated)	9 (30)
	Yes (self-identified or no confirmable credentials)	6 (20)
	No (layperson did not state medical affiliation)	13 (43)
	Medical clinic affiliated	2 (7)
**Evidence-based**		
	Yes	2 (7)
	No	28 (93)
**Cautionary message or disclaimer**		
	No	16 (53)
	Yes	14 (47)
**PEMAT^b^ understandability**		
	High (≥67%)	14 (47)
	Low (≤66%)	16 (53)
**PEMAT actionability**		
	High (≥67%)	15 (50)
	Low (≤66%)	15 (50)
**Religious reference**		
	Yes	6 (20)
	No	24 (80)
**GQS^c^**		
	1 (very poor)	1 (3)
	2 (poor)	5 (17)
	3 (moderate)	7 (20)
	4 (good)	17 (60)
	5 (excellent)	0 (0)

^a^HPV: human papillomavirus.

^b^PEMAT: Patient Education Materials Assessment Tool.

^c^GQS: Global Quality Scale.

**Table 2:** Other characteristics of TikTok videos (N=30).

Engagement	Minimum-maximum	Median (IQR)
Like count	524-116,493	3062 (1370-18,629)
Share count	37-36,403	751.5 (199-4019)
View count	59,116-8,490,149	176,391 (92,166-592,176)

### Emotional Tone and Linguistic Register

Casual was the most common (16/30, 53%) code, followed by serious (8/30, 27%) and emotional (6/30, 20%). Emotional videos were more engaging than others, receiving the highest median likes and share counts. However, emotional videos were associated with lower global quality scores (median 2, IQR 2-3), while serious and casual videos received higher scores (median 4, IQR 4-4). The difference in GQS across tones was statistically significant (*P*=.01), indicating that higher engagement did not correspond with higher content quality.

### Content Types

The most common content was diet and alternative therapies (15/30, 50%), including content promoting the use of raw garlic, black seeds, or reducing hydrogenated oils. This was followed by screening and early detection (6/30, 20%) and HPV vaccination (3/30, 10%). Other content types, including self-examination, stress, smoking cessation, and survivor stories, each appeared in only 1 of 30 (3%) videos. The videos in the top 25% (N=30) most-viewed videos focused on HPV vaccination, and those that mentioned cervical cancer were created by self-identified doctors, including 2 verified and 1 unverified account. Two used a serious tone, and 1 used a casual tone. All 3 videos received a global quality score (GQS) of 4.

### Cancers Mentioned

The most frequently mentioned cancers were general cancer (16/30, 53%), with breast cancer (5/30, 17%) and cervical cancer (4/30, 13%) commonly referenced. Less frequently mentioned were colon (2/30, 7%), bladder (1/30, 3%), multiple cancers (1/30, 3%), and testicular cancer (1/30, 3%).

### Speaker (Doctor, Self-Identified, or Layperson)

Among the top 25% (N=30) most-viewed videos, 17 (57%) were led by individuals identifying as doctors, including 9 (30%) verified doctors, 6 (20%) unverified, and 2 (7%) accounts affiliated with medical clinics. The remaining 13 of 30 (43%) accounts were led by laypeople. Of the 17 doctor-led accounts, 8 (47%) verified and 2 (12%) unverified accounts met the threshold for influencer status. Among the 13 laypeople accounts, 4 (31%) accounts met the influencer criteria. While only 4 of the 13 nondoctor-led accounts met the influencer threshold (≥100,000 followers), 3 additional laypeople’s accounts had a substantial following between 20,000 and 60,000 followers.

### Target Demographics, Religious Reference, and Presence of Cautionary Message

Religious framing appeared in 6 of 30 (20%) videos, with references to divine (*qadr*) will or spiritual health advice. Further, 14 of the 30 (47%) videos included a cautionary message or disclaimer, such as warnings about misinformation or clarification on cancer risk factors. Most videos targeted both genders (19/30, 63%), followed by women (8/30, 27%), young people (2/30, 7%), and men (1/30, 3%).

### Evidence-Based, Patient Education Materials Assessment Tool: Understandability and Actionability

Only 2 (7%) of the top 25% (N=30) most-viewed videos explicitly cited scientific literature or guidelines. A total of 53% (16/30) of videos were rated low on understandability (score ≤66%). Notably, all 6 doctor-led videos promoting diet and alternative therapies scored high (≥67%) for understandability, while all 9 layperson videos in that category scored low. Furthermore, 50% (15/30) of videos were rated low on actionability. Among diet and alternative health-related videos, those led by doctors were more likely to be actionable by 83% (25/30) compared to those led by laypeople (17/30, 56.6%), though this difference was not statistically significant (*P*=.58).

### About GQS

A total of 60% (18/30) of videos were rated as good (score of 4), 23% (7/30) as moderate (score of 3), and 17% (5/30) as poor (score of 2). Only 1 (3%) video was rated very poor (score of 1), and none were rated excellent. Videos led by doctors promoting diet and alternative therapies had significantly higher GQS scores than those led by laypeople (*P*=.06).

### Human and AI Agreement

Agreement between human coders was high across all domains (κ=0.84). There was perfect agreement between human and AI annotations for cancer type (κ=1.0) and strong agreement for GQS scoring (κ=0.94), though most discrepancies occurred between scores of 3 (moderate) and 4 (good), indicating difficulty distinguishing between mid and high-quality content. Agreement was lower for tone classification (κ=0.15), with AI misclassifying emotional delivery when relying on text-based input alone.

### Geographic Reach of Arabic-Language Cancer Prevention Content on TikTok

TikTok platform data indicated that Arabic-language cancer prevention content generated substantial engagement from users in both Arab-majority countries (eg, Egypt, Jordan, and Saudi Arabia) and diaspora contexts such as the United States, France, and Germany. The United States ranked in the top 10 for total views, highlighting the global reach of Arabic-language cancer messaging.

There was high agreement in cancer type between human and AI annotations (κ=1.0), and similarly high agreement in GQS scoring (κ=0.94). Tone classification showed lower concordance. While the AI model correctly identified many casual and serious tones, it misclassified emotional content in several cases, resulting in slight overall agreement (κ=0.15). Manual review confirmed that GPT-based annotation performed reliably across dialectal variations from multiple Arabic-speaking countries, indicating that cross-dialect consistency is achievable when coupled with human verification.

## Discussion

### Principal Findings

This study makes 3 contributions to the broader literature on online health videos and TikTok specifically. First, it provides the first systematic analysis of Arabic-language TikTok videos on cancer prevention. Second, it identifies an engagement quality gap in Arabic language cancer prevention content, extending prior English-language findings that emotionally charged posts often receive higher engagement [38]. Third, the present study advances methodological research by evaluating GPT-4’s performance on Arabic transcript-only inputs from short-form videos: the model demonstrated high reliability for structured categorical variables but low reliability for tone classification, underscoring the need for multimodal approaches that incorporate audio and visual cues. Together, these contributions deepen the understanding of Arabic-language TikTok health communication and illustrate both the potential and current limitations of AI-assisted content analysis across global digital ecosystems. While prior work has shown that LLMs can support qualitative researchers by generating themes from social media corpora in a single prompt [39], their use for systematic content analysis of short-form video data has not, to our knowledge, been previously demonstrated.

Within our sample, videos promoting diet and alternative therapies were among the most viewed. Studies in other cultural contexts have similarly shown that traditional or community-based health guidance often thrives because it is relational, linguistically resonant, and perceived as more trustworthy than institutional messages [40]. Research on TikTok in English more broadly echoes this pattern: a content analysis of health-related “EduTok” videos found that audiences most frequently engaged with educational posts related to diet, exercise, and sexual health, suggesting consistent user interest in familiar, lifestyle-oriented themes [41]. Together, these parallels suggest that what circulates widely on Arabic TikTok may reflect a broader sociocultural logic in which familiarity and affective connection drive credibility and engagement. It is important to note, however, that patterns related to content type and cancer type in this study are driven primarily by a small number of highly represented categories (eg, diet and alternative therapies and screening and early detection), which reflects the distribution of high-engagement Arabic-language TikTok content rather than a comprehensive representation of cancer prevention topics.

Beyond these general patterns, our data illustrate how an engagement quality gap appears specifically within Arabic TikTok cancer prevention content. Emotional tone was associated with higher engagement, even when informational quality was low. One widely circulated video, for example, claimed that “toxic people, not food or genetics,” cause cancer, an emotionally resonant but scientifically inaccurate claim that drew considerable engagement. These findings align with prior TikTok-specific studies showing that affectively charged content outperforms factual or instructional posts [41]. Importantly, our results do not imply that emotional tone alone determines virality; rather, they suggest that emotional framing, creator identity, and algorithmic amplification together create conditions in which lower-quality but more affectively engaging messages can spread widely.

Targeting of young people was limited despite TikTok’s prominence among youth. Only a small proportion of high-engagement videos explicitly addressed adolescents or young adults, even though early-life behaviors, such as HPV vaccination, tobacco use, diet, and physical activity, are critical for cancer prevention. The absence of youth-directed content suggests a missed opportunity to leverage TikTok as a public health tool for early prevention messaging. Instead, content often targeted adult women or general audiences, which may reflect creator demographics or cultural communication norms. However, it is important to recognize that reliance on TikTok for health information is not limited to adolescents. Many Arabic speakers in diaspora contexts, including Arab Americans, turn to social media due to linguistic and cultural barriers in traditional health care settings [17]. Consistent with this, our platform data showed high engagement from diaspora countries, including the United States, emphasizing TikTok’s role as a transnational source of Arabic language health information. Furthermore, prior research has shown that immigrants frequently rely on online platforms for relatable and accessible health content, making the quality of digital communication a critical equity issue [42]. These patterns parallel findings from US studies showing that African American and Hispanic adults were more likely than White adults to seek health information through social media during the COVID-19 pandemic, underscoring how communication inequities can drive platform reliance among marginalized groups [42]. Future research should examine whether these same patterns extend to Arabic-language health content on other short-form video platforms such as Instagram Reels (Meta), YouTube Shorts (Google LLC), Facebook Watch (Meta), and Snapchat Spotlight (Snap Inc), which share similar algorithmic dynamics but may differ in moderation and audience reach.

Most of the analyzed videos lacked references to peer-reviewed literature or established clinical guidelines, and only 30% (9/30) were led by doctors (whose credentials could be corroborated). It is important to distinguish between being evidence-based and citing sources. While a video may communicate content that aligns with scientific consensus, the absence of explicit references may reduce credibility, especially in digital environments where users rely on transparency to assess trustworthiness. Although doctor-led videos produced higher quality content on average (eg, higher GQS scores), professional identity alone did not ensure high understandability or actionability (high understandability suggests that most viewers, including those with limited health literacy, can grasp the essential messages being communicated). This is particularly salient given that populations with lower health literacy are more likely to rely on TikTok for health information [43].

Interpreting PEMAT AV and GQS together provides important insight into the quality of doctor-led content. PEMAT AV, which is validated for audiovisual materials, assesses whether information is communicated clearly and whether viewers are given actionable guidance, whereas GQS reflects a broader, more subjective appraisal of overall informational quality and usefulness. These differences are therefore meaningful rather than contradictory and underscore the value of using PEMAT AV and GQS as complementary measures when evaluating short-form health content [33]. Future studies may benefit from incorporating additional quality frameworks to further capture dimensions of informational rigor and communicative nuance.

Our findings show that doctor-led videos achieved higher quality scores but did not generate comparable engagement. This aligns with prior research on Arabic language health content across other platforms. For instance, studies of Arabic breast cancer videos on YouTube have shown that videos produced by trusted institutions tend to be more accurate but far less popular than those by individual users. This recurring pattern across platforms suggests that credibility alone does not guarantee visibility, a consistent challenge in health communication on social media. Effective health communication may therefore require pairing evidence-based content with narrative appeal, cultural resonance, and accessible delivery formats to compete with misinformation and emotionally engaging but lower-quality material.

Content moderation practices also play a role in shaping what health information circulates across Arabic-speaking regions. Unlike platforms that apply uniform global policies, TikTok relies on region-specific moderation teams and language filters, which may inconsistently flag or downrank health misinformation. While this study focused on cancer prevention, the engagement and credibility patterns we observed echo those reported in Arabic language TikTok vaccine content, suggesting that visibility dynamics may be shaped more by platform design and algorithmic incentives than by the specific health topic itself [22].

Finally, our study underscores the promise of LLMs for scalable analysis of health-related content in underrepresented languages. GPT-based coding achieved high reliability in classifying categorical variables such as cancer type and video quality (κ=0.94 to κ=1.0). However, it performed less consistently in detecting tone and register, particularly emotional delivery.

This finding stands in partial contrast to prior work, which has reported strong LLM performance in multilingual sentiment analysis of social media content [26]. A key difference, however, lies in the approach, which used large-scale labeled data from high-resource, text-based platforms. In contrast, our 1-shot method relied on transcripts of Arabic TikTok videos, where much of the emotional tone is conveyed through audiovisual cues such as intonation and facial expression not captured in text alone.

Emerging multimodal AI systems such as Gemini (Google LLC) and Google Cloud Video Intelligence, which can jointly interpret audio, visual, and textual inputs, hold promise for overcoming these limitations and enabling more contextually accurate annotation of short-form health content across languages. Applied tools such as ScreenApp, which automatically integrates speech-to-text, speaker detection, and scene-level video analysis, further illustrate how multimodal pipelines are already being used to extract meaningful patterns from audiovisual content [44].

The observed pattern in our findings points to 2 directions for future work: (1) developing a labeled Arabic TikTok dataset to refine tone detection in LLMs, and (2) adopting multimodal pipelines that combine audio, visual, and text cues to better capture emotion and on-screen gestures. This hybrid approach, using LLMs for large-scale triage and multimodal or human review for nuanced interpretation, offers a scalable path for improving health content analysis across languages.

These findings have important implications for public health outreach in Arabic-speaking communities, both within the Arab world and across diaspora populations. Given the limited availability of culturally tailored, Arabic-language health education materials and the growing reliance on social media for information, TikTok represents both a powerful tool and a potential vector for misinformation. While it can amplify accurate messaging, it also enables the rapid spread of misinformation. Addressing this will require multipronged strategies: empowering health care providers with the skills to create engaging content, leveraging AI for content monitoring, and partnering with trusted community figures to amplify reliable messages. Future interventions may leverage narrative and emotionally resonant formats to pair evidence-based content with styles that match user engagement preferences.

### Limitations

This study has several limitations. First, it focused on videos in the top 25% (N=30) most-viewed, which introduces an engagement bias and thus may not fully represent the broader landscape of Arabic-language cancer prevention content on TikTok. In addition, AI comparison was applied only to these top-viewed videos, rather than the full set of 320 eligible videos, limiting our ability to fully assess the scalability and generalizability of AI-based annotation across the entire dataset.

Second, we did not analyze or control for video length, which may influence engagement metrics. However, because the sample was drawn from the top 25% (N=30) of most-viewed videos, it likely reflects videos optimized for typical TikTok viewing behavior, minimizing major variability in duration effects. Third, while the Patient Education Materials Assessment Tool and GQS frameworks are validated tools, they may not fully capture the stylistic and communicative nuances characteristic of short-form, audiovisual social media content. Fourth, to facilitate AI-based classification, tone and linguistic register were simplified into broad categories, which may have limited the detection of the more subtle or culturally embedded communication styles typically captured through qualitative analysis. Fifth, although use of the TikTok API helped reduce algorithmic sampling bias, platform-specific ranking mechanisms and personalization features may still have influenced which videos achieved high visibility. In addition, analyses of content type and cancer type were concentrated within a narrow subset of categories due to the limited representation of other topics, and findings should therefore not be generalized to less frequently represented cancers or prevention behaviors. Finally, the AI model operated solely on transcribed audio, analyzing text without access to visual or prosodic cues such as facial expressions, gestures, or intonation, elements that are often common in video-based communication.

## Abbreviations

AI: artificial intelligence
API: application programming interface
GQS: Global Quality Scale
LLM: large language model
PEMAT AV: Patient Education Materials Assessment Tool for Audiovisual Materials
